# Comparison between HIV self-testing and facility-based HIV testing approach on HIV early detection among men who have sex with men: A cross-sectional study

**DOI:** 10.3389/fimmu.2022.857905

**Published:** 2022-09-13

**Authors:** Yi Zhou, Shaoli Huang, Mingting Cui, Zhihui Guo, Haotong Tang, Hang Lyu, Yuxin Ni, Ying Lu, Yunlong Feng, Yuyu Wang, Fengshi Jing, Shanzi Huang, Jiarun Li, Yao Xu, Wenhua Mei

**Affiliations:** ^1^ Department of HIV Prevention, Zhuhai Center for Disease Control and Prevention, Zhuhai, China; ^2^ Faculty of Medicine, Macau University of Science and Technology, Macao, Macao SAR, China; ^3^ Zhejiang University-University of Edinburgh Institute, Zhejiang University-University, Hangzhou, China; ^4^ School of Public Health (Shenzhen), Sun Yat-sen University, Shenzhen, China; ^5^ Dermatology Hospital of South Medical University, Guangzhou, China; ^6^ University of North Carolina Project-China, Guangzhou, China; ^7^ Department of Public Health and Preventive Medicine, School of Medicine Jinan University, Guangzhou, China; ^8^ Institute for Healthcare Artificial Intelligence, Guangdong Second Provincial General Hospital, Guangzhou, China; ^9^ School of Data Science, City University of Hong Kong, Hong Kong, Hong Kong SAR, China; ^10^ Department of Paediatrics, School of Clinical Science, Monash University, Melbourne, VIC, Australia

**Keywords:** HIV/AIDS, HIV self-testing, facility-based HIV testing, recent infection testing algorithms, RITA

## Abstract

**Background:**

To assess whether HIV self-testing (HIVST) has a better performance in identifying HIV-infected cases than the facility-based HIV testing (HIVFBT) approach.

**Methods:**

A cross-sectional study was conducted among men who have sex with men (MSM) by using an online questionnaire (including information on sociodemographic, sexual biography, and HIV testing history) and blood samples (for limiting antigen avidity enzyme immunoassay, gene subtype testing, and taking confirmed HIV test). MSM who were firstly identified as HIV positive through HIVST and HIVFBT were compared. Chi-square or Fisher’s exact test was used to explore any association between both groups and their subgroups.

**Results:**

In total, 124 MSM HIV cases were identified from 2017 to 2021 in Zhuhai, China, including 60 identified through HIVST and 64 through HIVFBT. Participants in the HIVST group were younger (≤30 years, 76.7% *vs.* 46.9%), were better educated (>high school, 61.7% *vs.* 39.1%), and had higher viral load (≥1,000 copies/ml, 71.7% *vs.* 50.0%) than MSM cases identified through HIVFBT. The proportion of early HIV infection in the HIVST group was higher than in the HIVFBT group, identified using four recent infection testing algorithms (RITAs) (RITA 1, 46.7% *vs.* 25.0%; RITA 2, 43.3% *vs.* 20.3%; RITA 3, 30.0% *vs.* 14.1%; RITA 4, 26.7% *vs.* 10.9%; all *p* < 0.05).

**Conclusions:**

The study showed that HIVST has better HIV early detection among MSM and that recent HIV infection cases mainly occur in younger and better-educated MSM. Compared with HIVFBT, HIVST is more accessible to the most at-risk population on time and tends to identify the case early. Further implementation studies are needed to fill the knowledge gap on this medical service model among MSM and other target populations.

## Introduction

In 2020, United Nations Member States committed to implementing 17 Sustainable Development Goals (SDGs), in which one of the motivations/goals is to end the AIDS epidemic by 2030 *via* using the Fast-Track approach with the 95-95-95 targets: by 2030, 95% of people living with HIV (PLWH) should know their HIV status, 95% of people who know their status should be on antiretroviral treatment (ART), and 95% of those on treatment should be virally suppressed (1–3). Acute and primary HIV infection, also known as early HIV infection (EHI), is the early stage of HIV infection. Acute HIV infection occurs 2 to 4 weeks after infection with both HIV RNA and p24 antigen present, and primary HIV infection occurs 6 months after infection. The high concentration of the virus during EHI leads to increased infectiousness, possibly as much as 26 times greater than during chronic infection (4). Improving the identification of EHI cases is important for HIV infection management to achieve the goal by 2030. However, in China, only 68% of people living with HIV were aware of their serology status by the end of 2015 (5). Only 15% of the identified cases are EHI in some high HIV prevalence provinces, which means most of the cases were diagnosed late (5). Thus, effective ways for early identification of EHIs are needed.

In the past years, several laboratory-based assays have been tested to identify early HIV infection according to the natural serological responses after infection (6). However, they were based on population data. When applied to individuals, false-positive results that classify long-term infection as a recent infection could occur. Thus, the World Health Organization (WHO) and the Joint United Nations Programme on HIV/AIDS (UNAIDS) recommend using recent infection testing algorithms (RITAs) to improve the accuracy of identifying recent HIV infections, which integrate HIV recency tests with multiple routinely used clinical assays (7). In the other words, a RITA is a combination of laboratory tests used to classify an HIV infection as recent (recent infections were acquired generally within 4 to 12 months) (8) or long-term (9). The limiting antigen avidity enzyme immunoassay (LAg-EIA) is one of the widely used serological assays to identify EHI (10–12). CD4+ T-cell count and viral load (VL) test are the other two clinical assays used for the majority of RITAs to reclassify recent infections (7). In comparison to the sole use of serological assays for the classification of recent HIV infection, RITAs, which combine various clinical information with the HIV recency assay, have been proven to accurately classify recent infection cases and effectively reduce false recent rate (FRR) (7).

Voluntary counselling and testing (VCT) and provider-initiated testing and counselling (PITC) are the majority of HIV tests conducted within health facilities and still represent the most common testing approach in most countries (3, 13). However, facility-based HIV testing (HIVFBT) is not accessible to the most at-risk population on time and tends to identify the case late. Thus, we will need a decentralized tool to reach the most at-risk participants.

The WHO encourages HIV rapid tests for screening high-risk populations (3, 14). In July 2019, 77 countries adopted policies or guidelines for the implementation and support of HIV self-testing (HIVST) (15). In China, the demand for and acceptability of HIVST among men who have sex with men (MSM) had been high, and many local community-based organizations were working with health bureaus in piloting HIVST among MSM (16, 17). A recent study has demonstrated that a model of social media-based HIVST with a secondary distribution is feasible and acceptable among MSM in China (18). HIVST can be used to identify first-time testers, promote HIV case identification, and link to care (18).

The aim of this study was to evaluate whether HIVST can identify PLWH earlier than the facility-based method.

## Methods

### Study setting

A community-based project was conducted from January 2017 to September 2021 to collect data on newly diagnosed HIV cases using two different approaches, HIVST and HIVFBT, among MSM in Zhuhai, China. Zhuhai is one of the first sites to pilot HIVST among Chinese MSM. It is estimated that there are 17,000 MSM living in Zhuhai, of whom 7% are HIV positive (19, 20). For HIVST, Zhuhai Center for Diseases Control and Prevention (CDC) and Zhuhai Xutong Voluntary Services Center (short for Xutong), a gay community-led organization, initiated a social media-based online system in 2016 for MSM to apply for free dual HIV/syphilis self-testing kits *via* Xutong’s public WeChat account. WeChat is one of the major communication social media platforms used for messaging, public surveys, and monetary transactions in China. The Lingnan Community Health Service Center is one of the HIVFBT sites in Zhuhai and accounted for more than 90% of HIVFBT among MSM each year. Therefore, for HIVFBT, the Lingnan Community Health Service Center in Zhuhai was selected as an HIVFBT site for recruiting participants.

### Participants

Adults who were a) biologically identified as men, b) aged 18 years and over, c) newly diagnosed as HIV positive, d) ever had anal sexual contact with a same-gender partner, and e) agreed to provide blood samples in Zhuhai were eligible for participation. Participants who used HIVST were classified into the HIVST group. Participants who adopted HIV testing at Lingnan Community Health Service Center and had not applied for free dual HIV/syphilis self-testing kits *via* Xutong’s public WeChat account were classified into the HIVFBT group. In addition, participants whose results of CD4+ T-cell count and VL were both not uploaded to the case-reporting system until 1 month after confirmed HIV tests were excluded.

### Procedure

After all eligible participants provided written consent, they had to complete an online survey including information about sociodemographic characteristics (age, education, marital status, and residence), sexual biography (gender identity, sexual orientation, and sexual orientation disclosure), and HIV testing history. A blood sample of 5 ml was taken from each individual for HIV serological testing, stored in a collection tube without anticoagulants, and centrifuged at 3,000 rpm for 5–10 min to obtain a serum sample. Then serum samples were kept at −20°C until processing. Blood specimens were firstly confirmed by Western blotting (HIV 2.2 WB, Genelabs Diagnostics, Singapore). Confirmed HIV cases were also tested for CD4+ T-cell count and viral load. Serological LAg-EIA assay (Beijing Kinghawk Pharmaceutical Co., Ltd., Beijing, China) was used to classify recent infection status. All laboratory tests were conducted in Zhuhai CDC.

### Data management and analysis

All data analyses were conducted in SPSS 26.0 for Windows 10. Chi-square or Fisher’s exact test was used to compare LAg-EIA, sociodemographic characteristics, and sexual behaviors between the HIVST and HIVFBT groups. Each group was also classified into four subgroups using different RITAs for the same analyses above: RITA 1 used the LAg-EIA only; RITA 2 combined the LAg-EIA with CD4+ T-cell count; RITA 3 combined the LAg-EIA with viral load; RITA 4 used the LAg-EIA, CD4+ T-cell count, and viral load together. Univariate and multivariate logistic analyses were used to identify factors associated with recent HIV infection. Two-sided *p*-values <0.05 were considered statistically significant.

### Ethical statement

Permission to conduct the project was obtained from the Human Research Ethics Committee of Zhuhai CDC (Ethics documents ID No. [2021]08).

## Results

In total, 124 (99.2%) of the 125 eligible men agreed to participate. Of them, 60 (48.0%) were from the HIVST group, while the other 64 (51.2%) were from the HIVFBT group.

### Sociodemographic characteristics

Compared with the HIVFBT group, participants in the HIVST group tended to be younger (aged 30 years or under, 76.7% *vs.* 46.9%, *p* = 0.001) and well educated (>high school, 61.7% *vs.* 39.1%, *p* = 0.012) and have had higher baseline viral load (≥1,000 copies/ml, 71.7% *vs.* 50.0%, *p* < 0.001) (**Table 1**). Participants in both groups had similar marital status, ethnic groups, baseline CD4+ T-cell counts, and genotype.

**Table 1:** Sociodemographic characteristics of MSM diagnosed as HIV positive in Zhuhai, China.

Variables	HIVST (n = 60)	HIVFBT (n = 64)	Total (n = 124)	*P*-Value
*Age (years)*	26 (23–30)	31 (26–39)	28 (24–34)	0.001*
≤30	46 (76.7%)	30 (46.9%)	76 (61.3%)	
>30	14 (23.3%)	34 (53.1%)	48 (38.7%)	
*Marital status*				
Single	53 (88.3%)	48 (75.0%)	101 (81.5%)	0.086
Married	2 (3.3%)	9 (14.1%)	11 (8.9%)	
Separated or divorced	5 (8.3%)	7 (10.9%)	12 (9.7%)	
*Educational level*				
≤High school	23 (38.3%)	39 (60.9%)	62 (50.0%)	0.012*
>High school	37 (61.7%)	25 (39.1%)	62 (50.0%)	
*Ethnic group*				
Han	58 (96.7%)	58 (90.6%)	116 (93.5%)	0.275
Others	2 (3.3%)	6 (9.4%)	8 (6.5%)	
*CD4 count (cells/µl)*				
<200	9 (15.0%)	14 (21.9%)	23 (18.5%)	0.305
≥200	51 (85.0%)	49 (76.6%)	100 (80.6%)	
Missing	–	1 (1.6%)	1 (0.8%)	
*Viral load (copies/ml)*				
<1000	17 (28.3%)	20 (28.1%)	37 (29.8%)	<0.001*
≥1,000	43 (71.7%)	34 (50.0%)	77 (62.1%)	
Missing	–	10 (21.9%)	10 (8.1%)	
*Genotype*				
CRF07_BC	23 (38.3%)	23 (35.9%)	46 (37.1%)	0.642
CRF01_AE	12 (20.0%)	10 (15.6%)	22 (17.7%)	
Others	12 (20.0%)	19 (29.7%)	31 (25.0%)	
Missing	13 (21.7%)	12 (18.8%)	25 (20.2%)	

*P*-Values were measured by chi-square test; p-value of CD4 count was measured by Fisher’s exact test. Age data are presented as median (25%–75%), and all other data are presented as n (%).

HIVST, HIV self-testing; HIVFBT, facility-based HIV testing; MSM, men who have sex with men.

*P < 0.05.

### Identification of recent HIV infection cases using different recent infection testing algorithms

**Figure 1:**
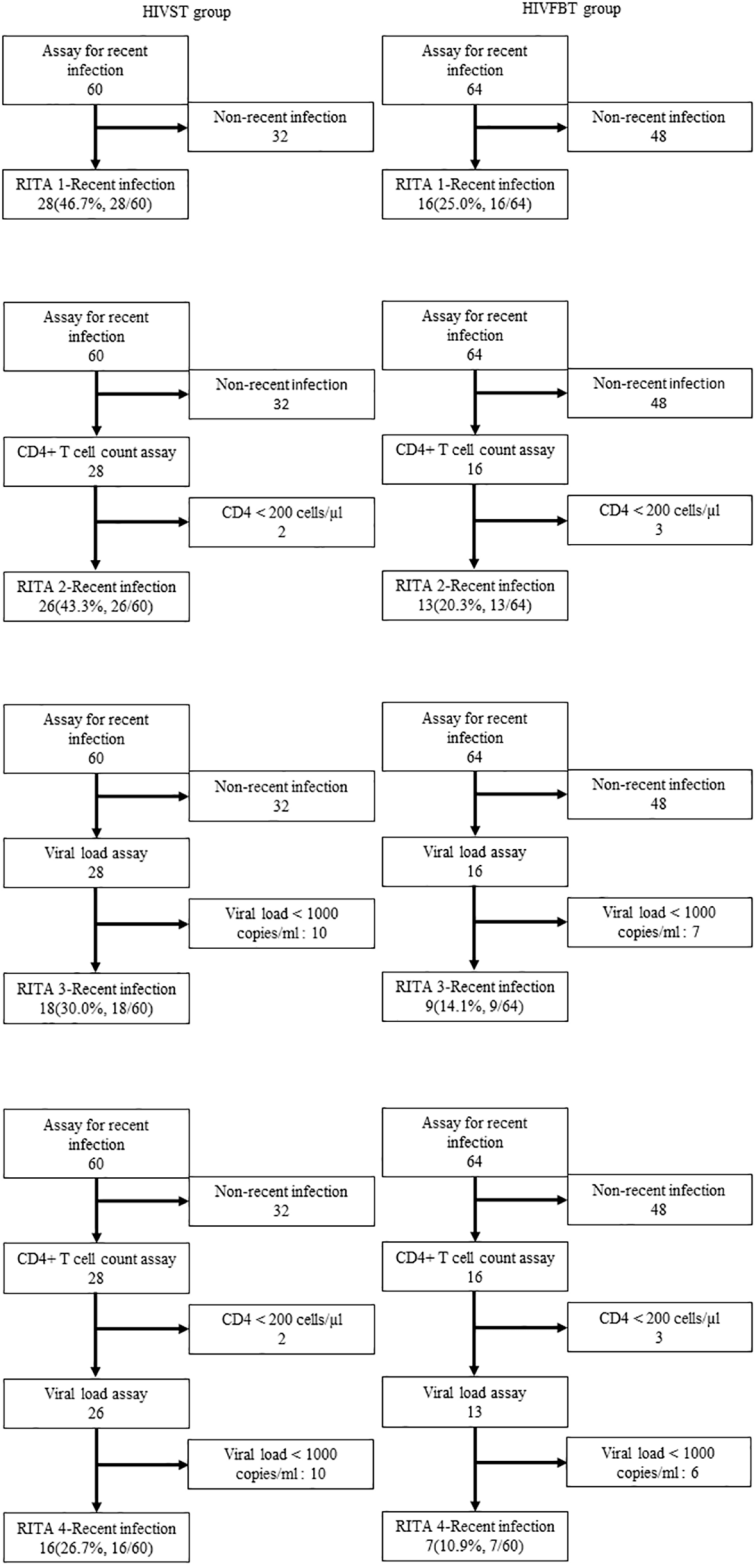
Flowchart of different recent infection testing algorithms (RITAs) among HIV infection status among men who have sex with men (MSM) diagnosed as HIV positive in Zhuhai, China. HIVST, HIV self-testing; HIVFBT, facility-based HIV testing; RITA 1, LAg-EIA only; RITA 2, LAg-EIA and CD4+ Tcell count; RITA 3, LAg-EIA and Viral Load; RITAs 4, LAg-EIA, CD4+ T cell count and Viral Load.

Four RITAs were implemented to classify recent HIV infection cases between the HIVST group and the HIVFBT group (**Figure 1**). According to the results of all RITAs, recent infectious cases were significantly associated with HIVST (**Table 2**). RITA 1 classified 28 (45.9%) recent infection cases from the HIVST group and 16 (25.0%) from the HIVFBT group with a strong significant difference. In RITA 2, two cases from the HIVST group were reclassified as non-recent due to CD4+ T-cell count <200 cells/µl, and three cases from the HIVFBT group were reclassified as non-recent due to CD4+ T-cell count <200 cells/µl. Together, RITA 2 classified a significant difference between the HIVST group and the HIVFBT group as 26 (42.5%) versus 13 (20.3%) cases, respectively. In RITA 3, 10 cases from the HIVST group were reclassified as non-recent due to VL < 1,000 copies/ml, and seven cases from the HIVFBT group were reclassified as non-recent due to VL < 1,000 copies/ml. The HIVST group (18, 29.5%) was significantly different from the HIVFBT group (9, 14.1%) when RITA 3 was used. RITA 4 combined all three assays and reclassified 44 cases in total as non-recent according to the exclusion criteria of CD4+ T-cell count and viral load (30 cases excluded for each) from the HIVST group and 57 cases from the HIVFBT group. RITA 4 identified significantly different numbers of recent infection cases in two groups, 16 (26.7%) from the HIVST group and seven (10.9%) from the HIVFBT group.

**Table 2:** HIV infection status among MSM diagnosed as HIV positive using different recent infection testing algorithms (RITAs) in Zhuhai, China.

Groups		HIVST (n = 60)	HIVFBT (n = 64)	*P*-Value
RITA 1	Recent infectious cases	28 (46.7%)	16 (25.0%)	0.012
	Non-recent infectious cases	32 (53.3%)	48 (75.0%)	
RITA 2	Recent infectious cases	26 (43.3%)	13 (20.3%)	0.006
	Non-recent infectious cases	34 (56.7%)	51 (79.7%)	
RITA 3	Recent infectious cases	18 (30.0%)	9 (14.1%)	0.032
	Non-recent infectious cases	42 (70.0%)	55 (85.9%)	
RITA 4	Recent infectious cases	16 (26.7%)	7 (10.9%)	0.024
	Non-recent infectious cases	44 (73.3%)	57 (89.1%)	

HIVST, HIV self-testing; HIVFBT, facility-based HIV testing; RITA 1, LAg-EIA only; RITA 2, LAg-EIA and CD4+ T-cell count; RITA 3, LAg-EIA and viral load; RITA 4, LAg-EIA, CD4+ T-cell count and viral load; MSM, men who have sex with men.

In the RITA subgroups, the sociodemographic characteristics of recent HIV infection cases between the HIVST and HIVFBT groups did not show significant differences (**Table 3**): more than half of the participants were aged 30 years or under and graduated from high school, and over 80% of the men were single and of Han ethnicity.

**Table 3:** Sociodemographic characteristics among MSM diagnosed as HIV positive with recency testing results using different recent infection testing algorithms (RITAs) in Zhuhai, China.

Variables	RITA 1 recent N (%)			RITA 2 recent N (%)			RITA 3 recent N (%)			RITA 4 recent N (%)		
	HIVST	HIVFBT	*P*-Value	HIVST	HIVFBT	*P*-Value	HIVST	HIVFBT	*P*-Value	HIVST	HIVFBT	*P*-Value
*Age (years)*												
≤30	22 (78.6%)	11 (68.8%)	0.492^a^	20 (76.9%)	10 (76.9%)	1.000^a^	13 (72.2%)	6 (66.7%)	1.000^a^	11 (68.8%)	5 (71.4%)	1.000^a^
>30	6 (21.4%)	5 (31.3%)		6 (23.1%)	3 (23.1%)		5 (27.8%)	3 (33.3%)		5 (31.3%)	2 (28.6%)	
*Marital status*												
Single	24 (85.7%)	15 (93.8%)	0.783^a^	22 (84.6%)	12 (92.3%)	0.789^a^	14 (77.8%)	8 (88.9%)	0.782^a^	12 (75.0%)	6 (85.7%)	1.000^a^
Married	2 (7.1%)	–		2 (7.7%)	–		2 (11.1%)	–		2 (12.5%)	–	
Separated or divorced	2 (7.1%)	1 (6.3%)		2 (7.7%)	1 (7.7%)		2 (11.1%)	1 (11.1%)		2 (12.5%)	1 (14.3%)	
*Educational level*												
≤High school	11 (39.3%)	7 (43.8%)	0.772^b^	10 (38.5%)	6 (46.2%)	0.645^b^	9 (50.0%)	5 (55.6%)	1.000^a^	8 (50.0%)	4 (57.1%)	1.000^a^
>High school	17 (60.7%)	9 (56.3%)		16 (61.5%)	7 (53.8%)		9 (50.0%)	4 (44.4%)		8 (50.0%)	3 (42.9%)	
*Ethnic group*												
Han	28 (100.0%)	14 (87.5%)	0.127^a^	26 (100.0%)	12 (92.3%)	0.333^a^	18 (100.0%)	8 (88.9%)	0.333^a^	16 (100.0%)	6 (85.7%)	0.304^a^
Others	–	2 (12.5%)		–	1 (7.7%)		–	1 (11.1%)		–	1 (14.3%)	
*Genotype*												
CRF07_BC	12 (42.9%)	4 (25.0%)	0.651^a^	12 (46.2%)	4 (30.8%)	0.681^a^	8 (44.4%)	2 (22.2%)	0.695^a^	8 (50.0%)	2 (28.6%)	0.716^a^
CRF01_AE	6 (21.4%)	4 (25.0%)		5 (19.2%)	2 (15.4%)		4 (22.2%)	2 (22.2%)		3 (18.8%)	1 (14.3%)	
Others	6 (21.4%)	4 (25.0%)		6 (23.1%)	4 (30.8%)		4 (22.2%)	3 (33.3%)		4 (25.0%)	3 (42.9%)	
Missing	4 (14.3%)	4 (25.0%)		3 (11.5%)	3 (23.1%)		2 (11.1%)	2 (22.2%)		1 (6.3%)	1 (14.3%)	

HIVST, HIV self-testing; HIVFBT, HIV facility-based testing; RITA 1, LAg-EIA only; RITA 2, LAg-EIA and CD4+ T-cell count; RITA 3, LAg-EIA and viral load; RITA 4, LAg-EIA, CD4+ T-cell count and viral load; MSM, men who have sex with men.

a Fisher’s exact probability test.

b Pearson chi-square test.

### Factors associated with recent HIV infection

Univariate and multivariate logistic analyses of factors associated with recent HIV infection are presented in **Table 4**. In the univariate analysis, recent HIV infections were more likely in the HIVST group (odds ratio (OR) 2.96, 95% confidence interval (CI) 1.12, 7.82). In the multivariate logistic model, HIVST had a statistically significant association with recent HIV infection (adjusted odds ratio (AOR) 3.14, 95% CI 1.08, 9.14).

**Table 4:** Univariate and multivariate logistic analyses of factors associated with recent HIV infection by RITA 4 (N = 124).

Variables	Non-recent infection (N = 101, %)	Recent infection (N = 23, %)	Univariate analysis	Multivariate analysis
			OR (95% CI)	AOR (95% CI)
*Group*					
HIVFBT	57 (56.4)	7 (30.4)	1	1	
HIVST	44 (43.6)	16 (69.6)	2.96 (1.12, 7.82) *	3.14 (1.08, 9.14) *	
*Age (year)*					
≤30	60 (59.4)	16 (69.6)	1	1	
>30	41 (40.6)	7 (30.4)	0.64 (0.24, 1.69)	0.52 (0.13, 1.99)	
*Marital status*					
Single	83 (82.2)	18 (78.3)	1	1	
Married	9 (8.9)	2 (8.7)	1.03 (0.20, 5.15)	1.96 (0.25, 15.61)	
Separated or divorced	9 (8.9)	3 (13.0)	1.54 (0.38, 6.25)	2.82 (0.44, 18.08)	
*Educational level*					
≤High school	50 (49.5)	12 (52.2)	1	1	
>High school	51 (50.5)	11 (47.8)	0.90 (0.36, 2.23)	0.75 (0.26, 2.15)	
*Ethnic group*					
Han	94 (93.1)	22 (95.7)	1	1	
Others	7 (6.9)	1 (4.3)	0.61 (0.07, 5.22)	0.73 (0.07, 8.03)	
*Genotype*					
CRF01_AE	18 (17.8)	4 (17.4)	1	1	
CRF07_BC	36 (35.6)	10 (43.5)	1.25 (0.34, 4.54)	1.45 (0.37, 5.75)	
Others	24 (23.8)	7 (30.4)	0.39 (0.06, 2.38)	1.80 (0.41, 7.89)	
Missing	23 (22.8)	2 (8.7)	1.31 (0.33, 5.18)	0.39 (0.06, 2.59)	

OR, odds ratio; AOR, adjusted odds ratio; HIVST, HIV self-testing; HIVFBT, HIV facility-based testing; RITA, recent infection testing algorithm.

*p < 0.05.

## Discussion

To our knowledge, this is the first study to compare the effectiveness of early detection of HIV infection between HIVST and HIVFBT among MSM in China and the first to compare the HIV genotype differences between HIVST and HIVFBT. This study provided strong evidence of the importance and effectiveness of assessing the early detection of HIV by self-testing. Along with other findings from the same project, the results demonstrated that HIVST expanded the coverage of HIV testing and improved the possibility of identifying new HIV cases in the Chinese MSM sample (18, 21).

From the current study, it has been found that more new HIV infection cases were found *via* self-testing than HIVFBT. Another study in China using the same RITAs has similar results that HIVST could identify more cases in EHI among MSM than other methods (22, 23). This may be because HIVST enables individuals to perform HIV antibody tests privately (24). Therefore, HIVST overcomes HIV-related stigma and discrimination and has the potential to increase the uptake of testing by reducing the structural barriers associated with clinic attendance (25, 26). Furthermore, normally, self-testing is designed as a rapid and easy-to-operate method like finger stick HIV self-tests. In this way, the time and effort spent traveling and attending a clinic or hospital and the waiting period can be reduced greatly. Although this method may also provide less sensitivity than laboratory-based testing, the period from detectable seroconversion to the first positive screening using self-tests may be shortened, as self-tests could increase testing frequency (27, 28). This is further proved by another study from the same project that HIV self-test kits, which showed an increase not only in case identification among MSM but also in the coverage of HIV testing as HIVST, are an easier way for secondary distribution from target participants (18). Notably, the target participants as sexual health influencers were associated with encouraging more alters with less testing access to self-tests for HIV (29). These results and evidence have demonstrated that HIVST could be a promising approach for increasing the rate of HIV testing in the period of EHI.

Identified cases in EHI in this study mainly occur in younger and better-educated MSM between the HIVST and HIVFBT groups. The same trend was reported in HIV annual reports in China and worldwide (30, 31). In China, young MSM aged 18 to 29 have a higher risk of spreading HIV than heterosexuals of a similar age, and the trend keeps increasing (32–39). In the United States, young MSM aged 20 to 29 were more likely to have “any” sexually transmitted infection, because they were more likely to have HIV-discordant condomless receptive intercourse and avoid disclosing same-sex behavior to healthcare providers or delay HIV/STI diagnosis and treatment (40, 41). These results and evidence have demonstrated that HIVST could be a promising approach to reach more at-risk populations on time.

The current study also assayed the genotypes of EHI cases. The results have discovered similar proportions in the genotypes of the EHI case compared to those in other cities in the same province. For example, the first dominant HIV-1 subtype circulating among MSM in Guangzhou was CRF07_BC (41.6%), followed by CRF01_AE (30.0%), and the same was found in Shenzhen, with CRF07_BC (39.1%) and CRF01_AE (35.1%) being the most predominant (42, 43). HIV-1 subtypes are associated with the pathogenesis and progression of AIDS-defining illness in the infected host (44). A systematic review of the trend of disease progression among HIV-1 different subtypes indicated that HIV-1 genetic diversity did seem to affect the rate of disease progression in ART-naive patient populations (45). Therefore, the similar proportion of subtypes among different cities keeps the comparability on EHI and inspires the possibility of the implementation of future community- and societal-level interventions.

Several limitations have been noticed in this study. Firstly, the results of LAg-EIA are largely affected by ART (World Health Organization, 2018) (9), and although information on receiving ART was not able to be collected in this study, the participants were firstly diagnosed as HIV positive in Zhuhai, and they were less likely to receive ART. Secondly, CD4+ T-cell count or VL testing information was missing for some participants in the HIVFBT group mainly due to a lack of specimens, and thus some cases could not be reclassified. However, the missing data should not greatly influence the chance of a significant difference between the two groups, as the EHI cases were also evaluated by the RITA 4 approach. Thirdly, the limited number of patients included in this study might result in lower statistical power. Finally, this study was a cross-sectional study. Only the associations or differences between the subgroups in a certain period can be reported due to the nature of the study design. Hence, it is hard to provide any direct evidence regarding the causes of EHI.

## Conclusion

This study showed that HIVST improved HIV early detection among MSM. Young and better-educated MSM seem more vulnerable to HIV infection than others, but this may be because of more likelihood of using HIVST methods. Compared with HIVFBT, HIVST is more accessible to the most at-risk population on time and tends to identify the case early. Thus, HIVST will be an important promising tool to reach the most at-risk population. Further implementation studies are needed to fill the knowledge gap on this medical service model among MSM and other target populations.

## Data availability statement

The original contributions presented in the study are included in the article/supplementary material. Further inquiries can be directed to the corresponding authors.

## Ethics statement

The studies involving human participants were reviewed and approved by Human Research Ethics Committee of Zhuhai Center for Disease Control and Prevention. The patients/participants provided their written informed consent to participate in this study.

## Author contributions

YZ designed the study and wrote the manuscript in collaboration with SLH, MC, and ZG. HT, YF, YW, ZG and MC, YN, YL, FJ, SZH, and JL performed laboratory work and/or data analysis. All authors discussed the results and commented on the manuscript. All authors contributed to the article and approved the submitted version.

## Funding

This study received support from Medical Research Funding of Guangdong Province funds (A2021011).

## Acknowledgments

We appreciate the contributions from all study participants, CBO volunteers, and staff from Zhuhai Center for Disease Control and Prevention, Zhuhai Xutong Voluntary Services Center, and SESH Group.

## Conflict of interest

The authors declare that the research was conducted in the absence of any commercial or financial relationships that could be construed as a potential conflict of interest.

The editor declared a shared affiliation with the authors, YN and YL, at the time of review.

## Publisher’s note

All claims expressed in this article are solely those of the authors and do not necessarily represent those of their affiliated organizations, or those of the publisher, the editors and the reviewers. Any product that may be evaluated in this article, or claim that may be made by its manufacturer, is not guaranteed or endorsed by the publisher.
